# Interspecific variation in responses to microclimate by terrestrial isopods: implications in relation to climate change

**DOI:** 10.3897/zookeys.801.24934

**Published:** 2018-12-03

**Authors:** Mark Hassall, Anna Moss, Bernice Dixie, James J. Gilroy

**Affiliations:** 1 School of Environmental Sciences, University of East Anglia, Norwich, NR4 7TJ, UK University of East Anglia Norwich United Kingdom; 2 School of Social Sciences, University of Dundee, Dundee, DD1 4HN, UK University of Dundee Dundee United Kingdom

**Keywords:** Aggregation, CO_2_ emissions from soils, feeding behaviour, future rainfall patterns, life history traits, norms of reaction, response curves, soil animals

## Abstract

The importance of considering species-specific biotic interactions when predicting feedbacks between the effects of climate change and ecosystem functions is becoming widely recognised. The responses of soil animals to predicted changes in global climate could potentially have far-reaching consequences for fluxes of soil carbon, including climatic feedbacks resulting from increased emissions of carbon dioxide from soils. The responses of soil animals to different microclimates can be summarised as norms of reaction, in order to compare phenotypic differences in traits along environmental gradients. Thermal and moisture reaction norms for physiological, behavioural and life history traits of species of terrestrial isopods differing in their morphological adaptations for reducing water loss are presented. Gradients of moisture reaction norms for respiratory rates and thermal reaction norms for water loss, for a species from the littoral zone were steeper than those for species from mesic environments. Those for mesic species were steeper than for those from xeric habitats. Within mesic species, gradients of thermal reaction norms for aggregation were steeper for *Oniscusasellus* than for *Porcellioscaber* or *Armadilliumvulgare*, and moisture reaction norms for sheltering and feeding behaviours were steeper for *Philosciamuscorum* than for either *P.scaber* or *A.vulgare*. These differences reflect differences in body shape, permeability of the cuticle, and development of pleopodal lungs. The implications of differences between different species of soil animals in response to microclimate on the possible influence of the soil fauna on soil carbon dynamics under future climates are discussed. In conclusion a modelling approach to bridging the inter-disciplinary gap between carbon cycling and the biology of soil animals is recommended.

## Introduction

In this review we draw attention to an important contribution that soil biologists, in particular those who study the biology of terrestrial isopods, can potentially make to the current debate as to how global climate change may influence components of the global carbon cycle. Currently the greatest uncertainty in modelling the global carbon cycle is not the fluxes across the ocean atmosphere interface or fluxes relating to net primary production but in modelling carbon fluxes within the soil. Globally ten times more carbon dioxide is emitted from soils than from all anthropogenic sources combined (Adger and Brown 1995, IPCC 2013). Anthropogenic induced climate change has caused shifts in both temperature and rainfall patterns across a range of geographic scales (Rosenzweig et al. 2008). Climate models predict global surface temperatures will rise by 0.3–4.8 °C by 2100 (IPCC 2014). A major challenge of current ecological research is to determine how ecosystem processes will respond to future environmental conditions (Santotja et al. 2017). It is widely acknowledged that food webs play pivotal roles in carbon cycles, including emissions of carbon dioxide from soils under climate change but this is rarely considered in modelling carbon fluxes (Pelini et al. 2015), while understanding of organismic physiological responses under climate change remains very rudimentary (Schmitz 2013). One question that is currently being debated is the extent to which soil animals can be modelled grouped together in broad trophic categories or whether they need to be considered at a finer level of functional trait or taxonomic resolution, depending upon their individual responses to changes in micro-climate (Jiguet et al. 2011, Dixie et al. 2015). Terrestrial isopods potentially form a useful model system with which to address this question because their behaviour, physiology, and life histories have been so extensively studied.

**Figure 1.**
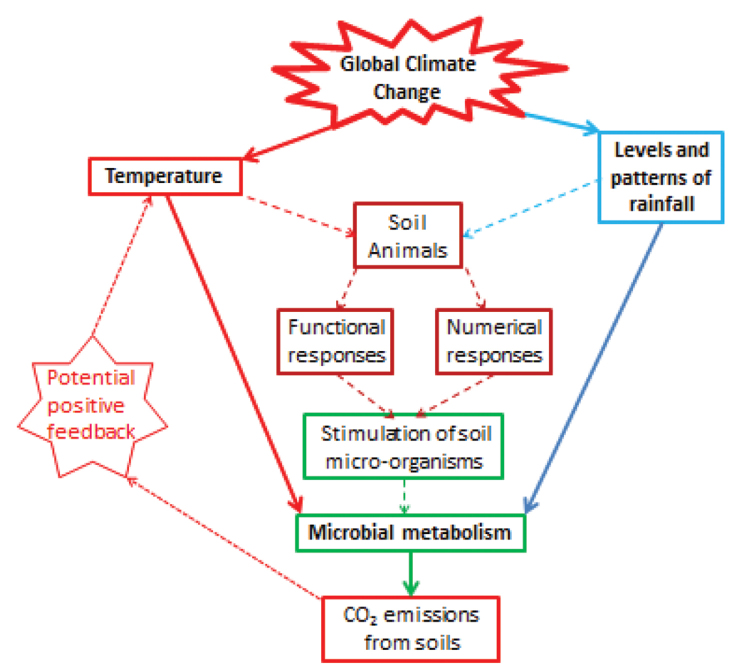
A conceptual diagram illustrating some of the pathways by which changes in global climate could potentially impact on rates of carbon dioxide emissions from soils. Both changes in temperature and in the levels and patterns of rainfall have strong direct effects on the metabolism of bacteria and fungi but their ecology and metabolism are also regulated by the extent to which they are stimulated by soil animals. Both functional (e.g., behavioural and physiological) responses and numerical (both life history and population) responses of soil animals are affected by their microclimate. This is in turn affected by larger scale changes in temperature and rainfall. Therefore, as well as their direct effect on microbial metabolism, these climatic variables have a strong indirect effect by influencing the behavioural, physiological, life history, and population processes of soil animals such as isopods.

Carbon dioxide emissions from soils are mediated predominately by microbial metabolism (Fig. 1), which, when not constrained by moisture availability, is a function of temperature (Schlesinger 1977). There is thus the potential for increased soil temperatures to accelerate emissions of carbon dioxide from soils. The further accumulation of CO_2_ in the atmosphere could then lead to further increases in temperature, leading to a positive feedback (Fig. 1) (Crowther et al. 2015, Thakur et al. 2018). This process is of most concern in regions with the highest pools of soil organic matter, such as tundra regions and temperate grasslands (Melillo et al. 2017). However, recent evidence indicates current carbon stores in other soils may also be changing from sinks to sources (Carrera et al. 2011) due to greater increases in soil respiration than in rates of carbon input to the soils.

The possible consequences of this positive feedback cycle could potentially be reduced if soil moisture were to decrease as a result of changes in rainfall patterns (Fig. 1). Climate models predict that globally rainfall will increase but it is predicted to vary strongly both spatially and temporally. Spatially, greater increases are projected for high latitudes and mid-latitude wet regions but decreases in subtropical dry regions and many mid-latitude regions (IPCC 2013). Temporally, summer rainfall in temperate regions, such as west and south Europe, is predicted to occur in fewer, but more intense episodes. These will result in longer periods of summer drought which could potentially constrain temperature-induced increases in soil carbon fluxes, including by reducing the stimulation of microbial metabolism by moisture dependent soil animals.

The microbially mediated emissions of carbon dioxide from soils are strongly influenced by soil animals acting as key system regulators (Fig. 1) (Anderson 1991, Lavelle and Spain 2001, Krab et al. 2010, Coleman et al. 2017). The significance of direct and indirect consequences of global climate change on ecosystem processes mediated by invertebrates, including their indirect effects on heterotroph respiration through interactions with microorganisms, are widely recognised (Pelini et al. 2015). However, although they are likely to have a major impact on ecosystems in the future, they are as yet understudied (Blankinship et al. 2011, Carrera et al. 2011, Del Toro et al. 2015).

It is becoming increasingly apparent that the importance of interspecific differences in key traits not only leads to different species having different functional roles (Dias et al. 2013, Zimmer et al. 2002) but also causes different species to be affected differently by climate change (Veldhuis et al. 2017). Because ecosystem science has yet to embrace how principles of evolutionary ecology explain the ways in which organisms mediate ecosystem carbon dynamics (Schmitz 2013), there is an urgent need to increase our understanding of how differential impacts of climate change on basic biological traits of individual species can lead to changes in community structure and function (Jiguet et al. 2011).

Traits of many soil animals are sensitive to relative humidity, making the soil fauna vulnerable to their activities being curtailed by changes in soil moisture (Krab et al. 2010, Crowther et al. 2015). Isopods are the most successful group of crustaceans to have made the transition to the terrestrial environment in terms of their abundance, distribution, and diversity, and have colonised a wide range of terrestrial habitats from the littoral zone to deserts (Edney 1954, Warburg 1987). They have become prominent members of the arthropod macro-decomposer guild in many ecosystems (Sutton 1980). Their morphology, physiology, behaviour, life histories, and ecology are known to be strongly influenced by micro-climates. Isopods therefore form good model soil animals for testing hypotheses concerning how inter-specific differences in their physiological, behavioural and life history traits will lead to interspecific differences in responses to predicted future changes in climate.

**Figure 2.**
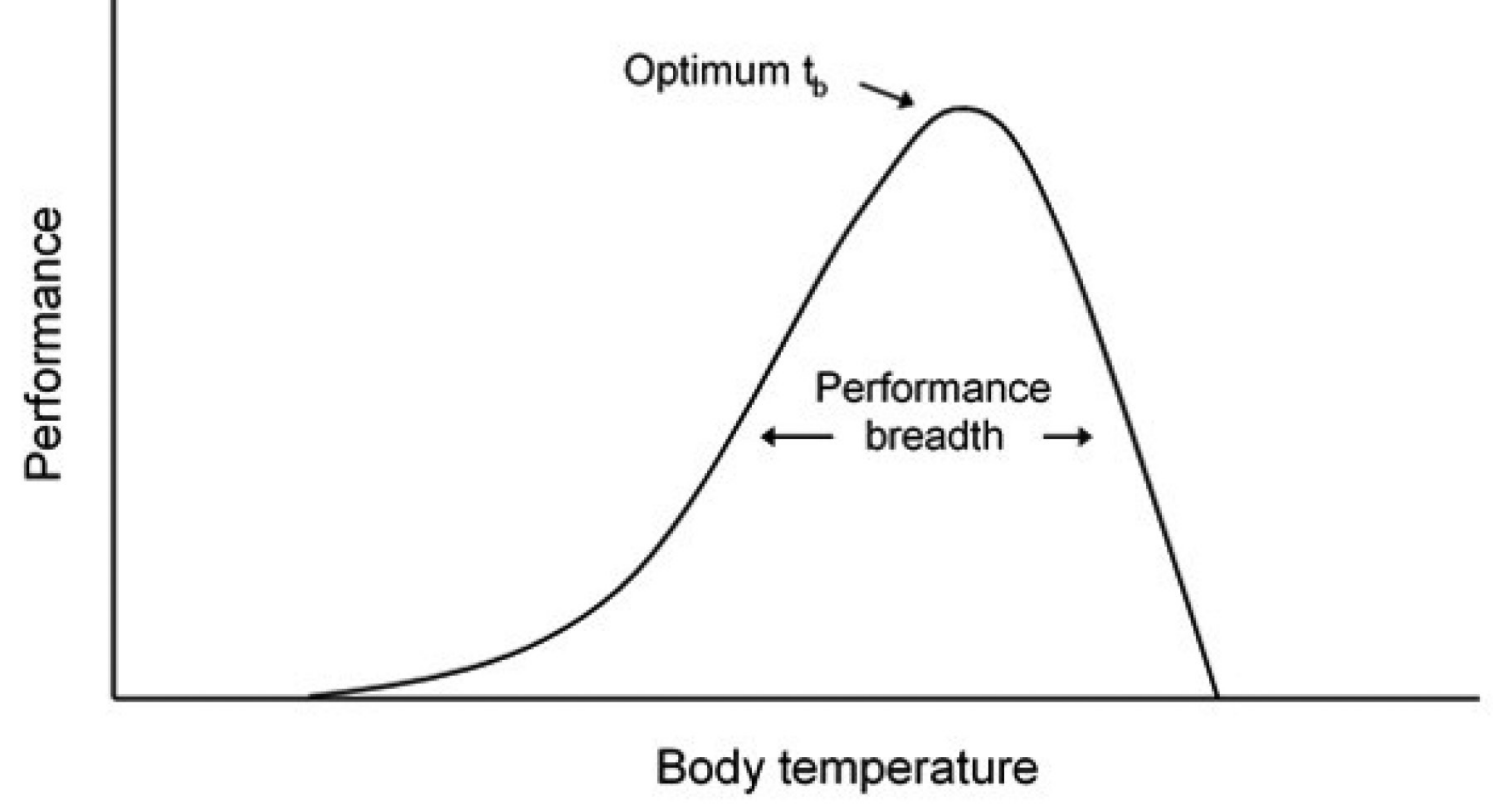
A schematic representation of a typical thermal response curve for enzymes (simplified from Huey and Kingsolver 1989). The temperature optimum is the temperature at which performance reaches its maximal level or peak performance. The performance breadth defines how steeply peaked (stenothermal) or broadly plateaued (eurythermal) the response curve is. Any part or the whole of such a curve can be considered to be a reaction norm of a genotype representing a range of phenotypes expressed across an environmental gradient, in this example, of temperature.

In this review we examine the extent to which traits of different species of terrestrial isopods known to have different strategies for reducing water loss in the terrestrial environment, respond to temperature and relative humidity. Phenotypic responses to a gradient in the environment can be summarised as norms of reaction. A reaction norm is the set of phenotypes produced in a range of environments (Schmalhausen 1949, Stearns 1992) and represents either the whole or part of a response curve of an organism to any environmental gradient as for example illustrated by the thermal response curve for enzymes shown in Fig. 2. Differences in the gradients of performance response curves, such as that in Fig. 2, denote how quickly an organism changes its performance for a given change in an environmental gradient. A steep gradient indicates a strong response and a high level of phenotypic plasticity over that region of the environmental gradient. Reaction norms can be very effective in comparing responses not just between different species as has been demonstrated very clearly by Dias et al. (2013) but also to different environmental gradients such as soil moisture and temperature.

We investigated differences in reaction norms of physiological, behavioural, and life history traits in response to differences in temperature and relative humidity for a range of isopod species chosen to reflect differences in their eco-morphology (Schmalfuss 1984, Schmidt and Wägele 2001, Broly et al. 2014) and their eco-physiology (Spencer and Edney 1954, Edney 1968, Warburg 1987, 1989, Hornung 2011). We used the selected species to explore possible implications of inter-specific differences in responses to micro-climate for the role of soil animals in soil processes, including regulation of microbially mediated soil carbon dynamics (Heemsbergen et al. 2004), under predicted climate change scenarios.

## Behavioural responses to microclimate: thermal norms of reaction for aggregating behaviour

*Case study 1.* Here we compare differences in aggregating behaviour of three species of isopod as described by Dixie et al. (2015) who give full details of the methodology including those of the statistical analyses used.

**Figure 3.**
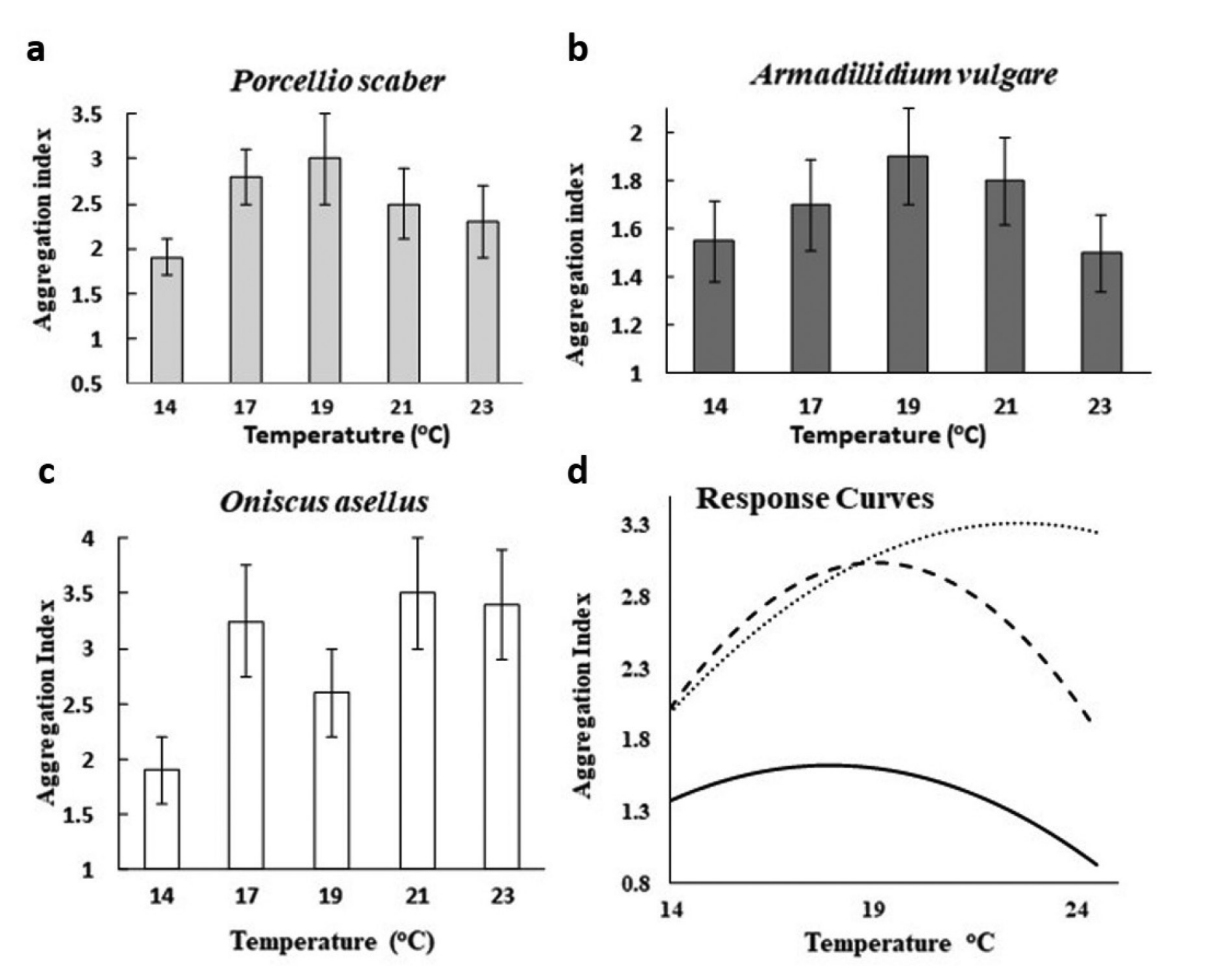
Aggregation of isopod species differing in desiccation resistance at different temperatures. Mean ± 1 SE aggregation indices (variance:mean ratio) at 90% relative humidity. **a***P.scaber* (F _4,249_ = 3.76, p < 0.01) **b***A.vulgare* (F _4,249_ = 1.97, P < 0.01) **c***O.asellus* (F _4, 249_ = 12.22, P < 0.001) **d** thermal reaction norms for aggregation expressed as quadratic response curves for: *P.scaber* (dashed line): y = -11.519 + 1.526× - 0.04×^2^; *A.vulgare* (solid line): y = -3.534 + 0.574× 0.016×^2^; *O.asellus* (dotted line): y = -5.890 + 0.814× – 0.018×^2^.

Differences in the degree of aggregation, at 90% relative humidity, between three species of isopods differing in their adaptations to reduce water loss in the terrestrial environment, are shown in Fig. 3. *Oniscusasellus* (Linnaeus, 1758) was most susceptible to water loss, the *Armadillidiumvulgare* (Latreille, 1804) is morphologically the best adapted to withstand desiccation while *Porcellioscaber* (Latreille, 1804) has an intermediate level of morphological adaptation but also uses aggregation as a key behavioural tactic in its overall desiccation avoidance strategy.

The temperature response curve of *P.scaber* (Fig. 3a, d) resembles the theoretical one in Fig. 2 in being an upwardly convex curve, with a peak in the aggregation index of 2.93 at 19 °C, a performance breadth at 90% of the peak of 4.7 °C and a gradient of the reaction norm from 14 °C to 17 °C of + 0.8 increase in aggregation index for this 3 °C rise in temperature. At temperatures higher than 19 °C the curve of the aggregation index decreases due to more of the animals leaving the clumps and moving around the arena sometimes climbing up the walls in an attempt to escape to find more favourable lower temperatures (Dixie et al. 2015).

*Armadillidiumvulgare* shows a broadly similar pattern of response (Fig. 3b, d), but with a lower peak of 1.9 in the aggregation index, again at 19 °C. The response curve is shallower than for *P.scaber* with a performance breadth of 6 °C at 90% of the peak value and a gradient for the reaction norm for the aggregation index between 14 -17 °C of +0.3. As for *P.scaber*, aggregation decreases at temperatures higher that the peak temperature.

The temperature response curve for aggregation of *O.asellus* (Fig. 3c, d) has a higher peak aggregation index of 3.35 at a higher temperature of 21.3 °C, a wide performance breadth of 6.2 °C at 90% of the peak value and a gradient for its thermal norm of reaction between 14 -17 °C of 1.5 in the aggregation index over this 3 °C range. This was approximately twice as steep as for *P.scaber* and three times as steep as for *A.vulgare*. The decline following the peak for *O.asellus* was less pronounced than for either of the other two species.

Overall these results show that these three species have substantially different patterns of thermal response curves for aggregating behaviours, reflecting differences in their morphological adaptations to terrestrial life.

## Behavioural responses to microclimate: moisture norms of reaction for sheltering and feeding behaviours

Many terrestrial isopods aggregate in shelter sites, particularly during the day, often under stones or pieces of wood where moisture from the soil maintains a more favourable relative humidity than in more open sites. They then emerge to forage at night when temperatures are lower and relative humidity is higher. Sheltering behaviour is thus of central importance in reducing mortality due to desiccation while also reducing the risk of being eaten by diurnal predators, such as insectivorous birds.

*Case study 2.* Effects of substrate moisture content and relative humidity on sheltering and feeding behaviour were investigated by Moss (2007) under controlled temperature conditions in the laboratory. Three species were studied: *A.vulgare*, *Philosciamuscorum* (Scopoli, 1763) and *P.scaber* differing in morphological and physiological adaptations to the terrestrial environment (Dias et al. 2012). The experimental mesocosms (220mm × 150mm × 80mm deep) were lined with plaster of Paris covered by 50mm sand with a feeding tray at one end and a shelter at the other, as described in further detail by Moss (2007). Two simulated rainfall regimes were used: high simulated rainfall, representing current mean summer daily rainfall intensity of 1.65 mm day^-1^ calculated from British Atmospheric Data Centre records for Lacock, Wiltshire, England (51°43'N, 2°11'W) during 1996–2006 when rainfall events occurred on average once every 72h. The low simulated rainfall regime was half the intensity of the high rainfall regime, 0.83 mm day^-1^, representing a scenario with 810 ppm concentration of atmospheric CO_2_ by 2080 which is predicted to result in a 50% reduction in summer rainfall in south west England (Hulme et al. 2002). For both regimes the simulated rainfall was administered at the start of each 72h period using a seedling watering can with 0.6 mm diameter holes in the rose with the shelter and food trays temporarily covered. Replicate arenas but without animals were used to monitor percentage moisture content of the substrate by taking samples of sand each morning and evening. Differences in the moisture conditions in the mesocosms were caused by both differences in intensity of simulated rainfall and the progressive drying out of mesocosms which occurred with time between rainfall events. Starting moisture level following high rainfall level ≈ 20% and for low rainfall level ≈ 10%.

Eight replicate mesocosms were used for each rainfall treatment, with six identical control boxes for monitoring substrate moisture content. Each mesocosm contained twenty individuals of each species. The behaviours of all animals over a 72-hour period were classified according to their location in the mesocosm and activity categories, including sheltering and presence in the feeding area. It was not possible to observe movements of mouthparts so time spent in the feeding area was assumed to be proportional to time spent feeding (Dias et al. 2012). Regression analyses of behavioural traits on substrate moisture were conducted.

**Figure 4.**
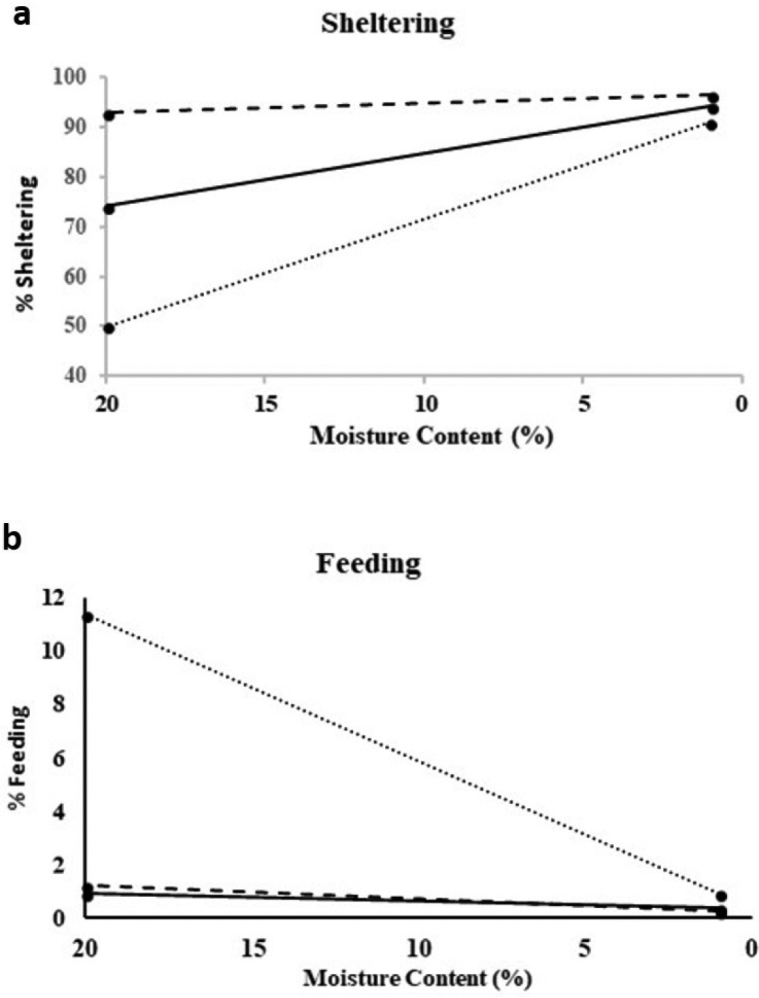
Moisture reaction norms for **a**) sheltering and **b**) feeding behaviours with changing sand moisture content (time spent in behaviour as percentages of total observed behaviours). Lines represent linear regression models: *A.vulgare* (solid line) (sheltering: y = 95.24 – 1.05×; feeding: y = 0.32 + 0.03), *P.scaber* (dashed line) (sheltering: y = 96.72 – 0.19×; feeding: y= 0.22 + 0.05×), *Ph.muscorum* (dotted line) (sheltering: y = 93.14 – 2.16×; feeding: y = 0.36 + 0.55x). Further regression statistics and number of observations (N), are given in Table 1.

The results in Fig. 4 and Table 1 show that *Ph.muscorum* spent significantly less time sheltering than either of the two other species under the highest moisture conditions. It also had the steepest gradient for its moisture reaction norm of -2.16, compared with values of -1.05 for *A.vulgare* and -0.19 for *P.scaber* (Table 1). *Porcellioscaber* showed the least change in the proportion of time it spent sheltering because it sheltered for more than 95% of the time under all substrate moisture conditions. In contrast *A.vulgare* and *Ph.muscorum* sheltered for 74% and 51% respectively of the time under the moistest conditions (Table 1).

*Ph.muscorum* also had the steepest gradient for its moisture reaction norm for feeding of 0.55 (Fig. 4b), which was significantly higher than those for either *P.scaber* (0.05) or *A.vulgare* (0.03) (Table 1). Time spent feeding decreased from 11.5% when moisture content of the sand was 20%, down to zero under the driest conditions when none of the three species spent a significant amount of time feeding.

Overall *Ph.muscorum* showed significantly steeper moisture reaction norms than did either of the other species just as *O.asellus*, which similarly lacks pleopodal lungs, had a steeper gradient for its thermal reaction norm for aggregation than did either *P.scaber* or *A.vulgare*.

**Table 1.** Regression statistics for moisture reaction norms for sheltering and feeding behaviours of three species of isopods, *Armadillidiumvulgare*, *Porcellioscaber*, and *Philosciamuscorum*, in laboratory arenas. Abbreviations: N: number of observations; *a* and *b*: parameters of regression equation; †: significant differences at P < 0.001(*t* test).

**Regression statistics**	**Sheltering**				**Feeding**		
	**N**	***a***	***b***	***t***	***a***	***b***	***t***
* A. vulgare *	1648	95.24	-1.05	-**21.84**†	0.32	0.03	**3.77**†
* P. muscorum *	824	93.14	-2.16	-**25.07**†	0.36	0.55	**15.32**†
* P. scaber *	1648	96.72	-0.19	-**8.39**†	0.22	0.05	**6.84**†
**Comparison of gradients**	**Sheltering**				**Feeding**		
*A.vulgare* vs. *P.muscorum*				**18.99**†			-**30.19**†
*A.vulgare* vs. *P.scaber*				-**25.08**†			-**3.96**†
*P.muscorum* vs. *P.scaber*				-**44.43**†			**28.36**†

## Life history responses to microclimate: thermal and moisture norms of reaction for growth and survivorship

*Case study 3*. Both thermal and moisture reaction norms for the key life history traits of growth and mortality were compared for *O.asellus* and *Porcelliodilatatus* Brandt, 1833 by Dixie et al. (2015) who give full details of the methodology and statistical analyses used.

The 2×2 factorial experimental design for investigating both relative growth rates and mortality rates permits comparison of thermal and moisture reaction norms simultaneously. Growth rates of *O.asellus* increased significantly at 5 °C higher temperatures under the drier, 70% relative humidity conditions but did not grow significantly faster at the higher temperatures in the moister, 90% humidity (Table 2). Growth of *P.dilatatus* increased with temperature to a similar extent under both experimental relative humidity conditions. All four of the moisture reaction norms for a 20% decrease in moisture are steeper than any of the thermal reaction norms for a 5 °C temperature difference for either species. This indicates a greater sensitivity to a 20% change in relative humidity than to a rise of 5 °C in temperature, suggesting that perhaps these species might respond more to predicted future changes in rainfall than to predicted increases in temperature. The difference in gradients of reaction norms between species (Table 2) reflects the higher rates of growth for *P.dilatatus* under all four combinations of temperature and relative humidity conditions.

Mortality rates (Table 3) were very low at 90% relative humidity; none of the *O.asellus* and very few of the *P.dilatatus* died at this relative humidity. The gradients of thermal reaction norms at 90% relative humidity were therefore not significantly different to zero. Under drier conditions, at 70% relative humidity, more individuals of both species died at 13.5 °C than at 18.5 °C, leading to steeper thermal reaction norms at 70% relative humidity than at 90% relative humidity.

Overall, 20% lower relative humidity resulted in steeper moisture reaction norms for mortality rates than for thermal reaction norms resulting from a 5 °C rise in temperature, with *O.asellus* being more susceptible to drier conditions than *P.dilatatus*.

**Table 2.** Relative growth rates (mg g^-1^ day^-1^) of *Oniscusasellus* and *Porcelliodilatatus*. Three way ANOVA: temperature F_1,72_ = 5.15, P = 0.026; humidity F_1,72_ = 88.62, P < 0.001; species F_1,72_ = 41.53, P < 0.001. Reaction norms are derived from the differences in response to the two temperature and two humidity conditions.

	Temperature °C	Relative humidity %		Moisture reaction norms
		90%	70%	
* O. asellus *	13.5 °C	34 ± 6	2 ± 0.1	-32
	18.5 °C	32 ± 2.5	17 ± 9	-15
	Thermal reaction norms	-2	15	
* P. dilatatus *	13.5 °C	66 ± 0.5	15 ± 4	-51
	18.5 °C	77 ± 8	28 ± 4	-49
	Thermal reaction norms	11	13	

**Table 3.** Mortality rates (numbers dying container ^-1^ 7days^-1^) of *O.asellus* and *P.dilatatus*. Mann Whitney U test for *O.asellus*: temperature NS, humidity U = 1851 P < 0.001; for *P.dilatatus*: temperature U = 2754, P = 0.016, humidity U = 2277, P < 0.001. Reaction norms are derived from the differences in response to the two temperature and two humidity conditions.

	**Temperature °C**	**Relative humidity** %		**Moisture reaction norms**
		**90**%	**70**%	
* O. asellus *	13.5 °C	0	0.73 ± 0.18	0.73
	18.5 °C	0	0.58 ± 0.12	0.58
	Thermal reaction norms	0	0.46	
* P. dilatatus *	13.5 °C	0.09 ± 0.04	0.78 ± 0.16	0.69
	18.5 °C	0.03 ± 0.03	0.18 ± 0.08	0.15
	Thermal reaction norms	0.06	0.60	

## Physiological responses to microclimate: temperature and moisture norms of reaction for respiratory rate and water loss

One of the drivers of differences between species of isopods in their behavioural and life history responses to differences in temperature and moisture is their different physiological adaptations to the terrestrial environment. In this section we compare responses of terrestrial isopod species, occurring in a wide range of biomes representing a gradient of moisture conditions.

Moisture reaction norms for differences in respiratory rate for *O.asellus*, *P.scaber*, and *A.vulgare* are shown in Fig. 4 together with that for *Ligiaoceanica* (Linnaeus, 1767). The pattern of differences between the physiological reaction norms closely parallels that of behavioural reaction norms (Figs 3, 4) with the gradient for *O.asellus* over a range of a 50% difference in relative humidity being substantially higher than for *P.scaber* and *A.vulgare*. However the gradient for *L.oceanica*, a littoral species, is more than double that for *O.asellus* reflecting the higher stress that would result from a reduction in relative humidity for a species that lives in a habitat with abundant availability of crevices with saturated micro-climates.

This interspecific comparison is extended for species from a wider range of habitats for thermal reaction norms for the physiological process of water loss in Fig. 6. The pattern for *L.oceanica*, *O.asellus* and *P.scaber* is similar to that for the moisture reaction norms for respiratory rate. *Ph.muscorum* has a higher rate than *O.asellus*, which, together with *P.scaber*, has a slightly steeper moisture reaction norm than either *A.vulgare* or *Armadillidiumnasatum* (Budde-Lund, 1885). All five of these species are found in mesic habitats. The three species of *Buddelundia* from semi-arid habitats in South Australia all have lower gradients for their water loss thermal reaction norms than any of the European species from mesic habitats. Least affected by differences in temperature are species from xeric habitats including those found in the Negev and North African deserts such as *Hemilepistusreaumurii* (Milne-Edwards, 1840). The two *Armadillo* species and *Porcellioolivieri* (Audouin, 1826) also occur in desert environments but not so exclusively, as they are also found in other dry Mediterranean habitats. These three species have gradients for their thermal reaction norms that are intermediate between those of the desert specialists (including *Venezilloarizonicus* Mulaik & Mulaik, 1942) and the mesic species.

## Discussion

**Figure 5.**
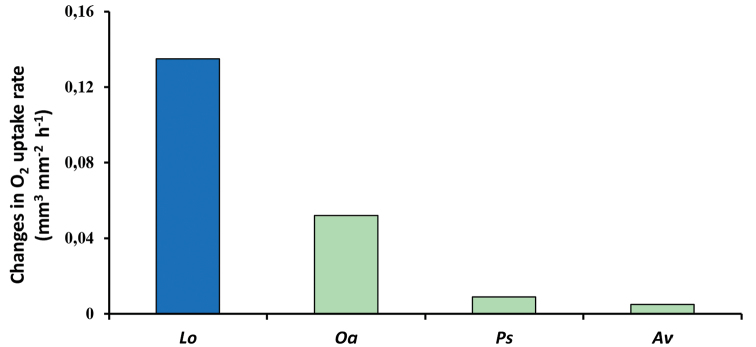
Gradients of moisture reaction norms for respiration of isopods differing in their resistance to desiccation. Reaction norms over the range 50–100% relative humidity for respiratory rates measured as rates of oxygen uptake (mm^3^ mm^-2^ body surface h^-1^) (Edney 1968). Key to species: *Lo Ligiaoceanica*, *Oa Oniscusasellus*, *Ps Porcellioscaber*, *Av Armadillidiumvulgare*. Key to habitats: littoral (blue), mesic (green).

The range of morphological adaptations in body shape, respiratory surfaces of the pleopods and cuticle of isopods have been comprehensively described (Edney 1968, Warburg 1992, Schmalfuss 1984, Hornung 2011, Csonka et al. 2013, Broly et al. 2014) and reflect a moisture gradient in the habitats in which they are found, from littoral, through mesic to xeric, including desert. It can therefore be predicted that there will be a parallel gradient in the magnitude of physiological responses to changes in microclimate reflected in the gradients of thermal and moisture reaction norms. This hypothesis was supported for respiratory rate moisture reaction norms (Fig. 5), as *L.oceanica*, the species with the most primitive morphological adaptations to the terrestrial environment, had the steepest reaction norm, while *O.asellus*, which does not have pleopodal lungs, had a steeper moisture reaction norm than either *P.scaber* or *A.vulgare*, both of which have well developed pleopodal lungs.

**Figure 6.**
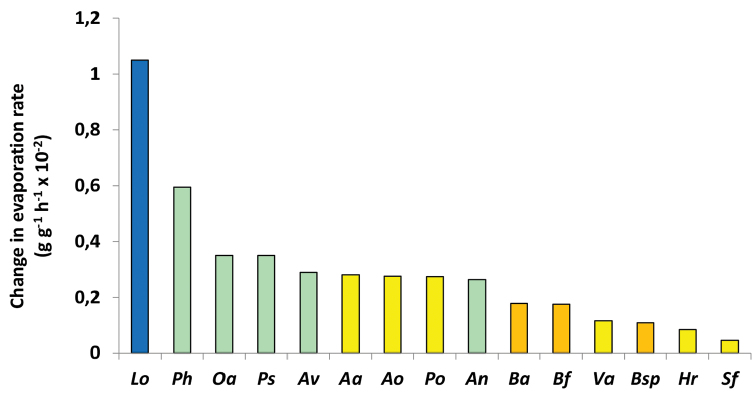
Thermal reaction norm gradients for evaporation rate (water loss) for isopods from biomes differing in availability of moisture. Evaporation rate (g g^-1^ h^-1^× 10^-2^) standardised to a temperature range of 3.5 °C (from Edney 1951; Warburg 1965, 1987, 1989). Key to species: *Lo Ligiaoceanica*, *Ph Philosciamuscorum*, *Oa Oniscusasellus*, *Ps Porcellioscaber*, *Av Armadillidiumvulgare*, *Ao Armadilloofficinalis*, *Aa Armadilloalbomarginatus*, *Po Porcellioolivieri*, *An Armadilliumnasatum*, *Ba Buddelundiaalbinogrisescens*, *Bf Buddelundiafrontosa*, *Va Veneziilloarizonicus*, *Bsp Buddelundia* spp. probably *lateralis*, *Hr Hemilepistusreaumurii*, *Sf Schizidiumfestai*. Key to habitats: littoral (blue), mesic (green), xeric (yellow), semi-arid (orange) habitats in South Australia.

Similarly, gradients of thermal reaction norms for water loss were substantially higher for *L.oceanica* than for the more fully terrestrial species *O.asellus* and *Ph.muscorum*, both of which had steeper gradients for their thermal reaction norms than members of either of the *Porcellio* species or *A.vulgare* (Fig. 6). There was a clear trend of thermal reaction norms for species from semi-arid environments in South Australia and xeric habitats in Europe, including specialist desert species, all having shallower thermal reaction norms for rates of water loss. Thus it can be predicted that those species from mesic environments will be more sensitive to changes in rainfall patterns, including longer periods of summer drought, than those from xeric habitats.

Given that these patterns of inter-specific differences in physiological traits for both thermal and moisture reaction norms reflect differences in morphology (Schmalfuss 1984, Broly et al. 2015), it can be predicted that there would also be a parallel pattern in thermal and moisture reaction norms for behavioural and life history traits. Aggregation in isopods is a behavioural tactic that reduces respiratory rates and water loss (Allee 1926), further elaborated by Friedlander (1965), Cloudsey-Thompson and Constaninou (1987), Caubet et al. (2008), and Broly et al. (2014). This morphologically related hypothesis is supported by both the peak of the thermal response curve and the gradient of the thermal reaction norm for aggregation being higher for *O.asellus* than for either *P.scaber* or *A.vulgare*.

Sheltering behaviour is another tactic evolved in isopods to help reduce water loss by taking refuge in more humid shelter sites. Again, *Ph.muscorum* had a significantly higher gradient for its sheltering moisture reaction norm than either *P.scaber* or *A.vulgare*. Similarly, feeding behaviour moisture reaction norms of *Ph.muscorum* decreased more steeply than for either of the other two species. Thus within three species, all typical in mesic environments, there was a consistent trend in all of these behavioural traits, with species without pleopodal lungs being more sensitive to changes in microclimate than species better adapted to resist desiccation. This again indicates that there are significant inter-specific differences which suggest those species least adapted to the terrestrial environment might be more susceptible to potential changes in micro-climate resulting from changes in future patterns of rainfall.

It would be logical to predict that such a trend might also apply to growth rates but that was not supported by the data because thermal reaction norms were very similar for *O.asellus* and *P.dilatatus* while the gradient for moisture reaction norms at both temperatures were higher for *P.dilatatus*. *P.dilatatus* had higher growth rates under all four combinations of temperature and relative humidity, possibly because the microclimate actually experienced by *P.dilatatus* was moister than that experienced by *O.asellus* due to *P.dilatatus* behavioural trait of burrowing into the sand substrates thereby being subject to a higher relative humidity in its immediate microclimate.

These life history traits of growth and survivorship are important correlates of fitness and have a very strong influence on the population dynamics of isopods. Differences in the gradients of reaction norms of life history traits could thus result in differences in the way abundances of these species might change under future climates.

An important conclusion of this overview is that, due to inter-specific differences in morphological, physiological, behavioural and life history traits, different species of isopods are likely to respond very differently to predicted changes in global climate. Interspecific differences in response to changes in temperature (Hickling et al. 2006) are now well documented for other taxa such as birds (Pearce-Higgins et al. 2015), Lepidoptera (McDermott Long et al. 2017), Orthoptera (Buckley et al. 2015) and Odonata (Stoks et al. 2014). In contrast, much less is known about ecological responses to predicted changes in rainfall patterns (Urban et al. 2016). Such responses are likely to be particularly pronounced for soil animals due to their activity and ecology being so strongly influenced by soil moisture, as shown by Blankinship et al. (2011) who found that precipitation limited all taxa and trophic groups in forest floors.

If the abundance of some species declines, it is not yet known whether other species, less sensitive to changes in microclimate, will respond by expanding their realised niches. It is known that interspecific competition for high quality foods between different species of isopods does occur in the field (Hassall and Dangerfield 1989) so it is possible that total guild densities may change less than species composition of guilds for macro-decomposers. It is known that differential impacts on basic biological parameters of individual species can modify fundamental characteristics of community structure and function (Jiguet et al. 2011). For isopods it is possible that the functional role of the whole isopod guild in stimulating microbial activity may alter less as a result of future climate change than might be expected from effects on particularly sensitive individual species. If other species in the guild, less sensitive to changes in micro-climate, expanded their realised niches in response to the decline of a more sensitive species, this could buffer consequences of climate change for overall decomposition rates. It is yet to be tested whether net community level responses, resulting from differential impacts of climate change on individual species, will impact on the extent to which future changes in rainfall patterns might mitigate potential increased temperature/ induced carbon dioxide emission feedbacks. It is widely agreed however, more focus is required on biotic interactions to clarify the potential feedbacks between climate change and soil carbon dynamics (Carrera et al. 2011).

The importance of species-specific responses to climate change has been highlighted for Collembola by Krab et al. (2010) and Makkonen et al. (2011), who found that interspecific faunal trait variation provided a valuable tool in predicting animal responses to climate change. However, it is noteworthy that Crowther et al. (2015) conclude that the regulatory effects of interspecific interactions are rarely considered in climate feedback studies. Pelini et al. (2015) extend this conclusion further by arguing that without isolating and including the significant impact of invertebrates, climate models will be incomplete, hindering well-informed policy decisions. Because the adaptive value of fundamental biological traits in isopods have been so comprehensively studied and because isopods form such a prominent component of so many soil macro-arthropod communities, we propose that terrestrial isopods represent excellent model systems for further investigation of species specific responses to predicted climate change and their consequences for soil carbon dynamics.

### What are the most pressing needs for future research in this field?

Our understanding of the global carbon cycle is predominantly encapsulated in models. Our understanding of terrestrial isopod biology, in contrast, is mostly based on results of empirical studies. There is a very important need to bridge the gap between these two contrasting approaches and methodologies in these completely different disciplines. Considering the whole series of symposium volumes on the Biology of Terrestrial Isopods from 1984 until 2018, models may qualify as an endangered species.

We know that carbon dynamics in the soil is the least well understood part of the global carbon cycle. Whilst, it is extremely complicated, models by definition are simplifications of reality. Models can never represent the full complexity of the real world, that is not their function, but what they have are both holistic and heuristic properties. Holistic in that it should be easier to appreciate emergent properties of a system as a whole from a model of it, rather than from detailed studies of individual components. Heuristic in that models should generate testable predictions that cannot be made on the basis of studying individual components in isolation.

Terrestrial isopods are soil animals about which we have a wealth of knowledge based on empirical studies on their anatomy, behaviour, physiology, life history, and ecology. What we do not have is integrative models of different aspects of their biology that can then be interfaced with those of other soil animals and ultimately with those of micro-organisms. This integrative approach would significantly contribute to our understanding of how global climate change will affect the soil component of the global carbon cycle.

### Where can we start?

Models do not necessarily have to be numerical, at least initially. A topographic map is a model of a landscape. At a glance, that piece of paper only tens of cm wide, shows us where hills, mountains, rivers and bridges are in landscapes at much larger scales. By using a map we can predict how best to travel from A to B. What is very urgently needed in soil biology is some comparably simplified maps of the interactions involving the soil animals under our feet. A starting place could be for the context of many future studies to be introduced using conceptual box and arrow models of the system under study (Fig. 1). Our skills as scientists lie in how effectively we can simplify the real world we are studying by being brave enough to make a whole array of untested assumptions.

The next stage could then be to parameterise these models using the extensive empirical data available in the literature, making assumptions we know to be simplistic but which enable us to make further quantitative predictions. Progressively testing these predictions experimentally to validate the models further could then increase our understanding of the system.

A problem will always be integrating models representing different levels of organisation which, from previous studies, range from molecules to communities. These may initially appear to be measured in different currencies but ultimately, while we are working within the paradigm of neo-Darwinian evolutionary theory, the answer to this problem may be in using fitness, or at least fitness correlates, to equate different traits at the individual level. Moving from the individual level to the ecosystem function level could then at least be based on a sound theoretical foundation.

The “functional traits” approach (Moretti et al. 2017) may be one way forward. Splitting whole trophic groups, treated as homogeneous entities in some large scale models, into functional groups has been an important step in the right direction. Taking account of interspecific differences in traits within functional groups and incorporating the diversity of responses into systems models could be a further important step. It would appear that the new generation of isopod biologists is now uniquely well placed to do this, thus giving them the potential to fill an extremely important gap in 21^st^ Century science in a way that is a meaningful contribution to our understanding of the effects mankind is having by interfering with the global carbon cycle and thus global climate systems. We wish younger colleagues all success with such a challenging venture at such an exciting time and best wishes with carrying the science of terrestrial isopod biology to new horizons.
